# Report of the Parana coffee root-knot nematode, *Meloidogyne paranaensis* (Tylenchida: Meloidogynidae) from *Caladium* sp. in the continental United States

**DOI:** 10.21307/jofnem-2021-108

**Published:** 2022-01-04

**Authors:** Sergei A. Subbotin, Julie Burbridge

**Affiliations:** 1 Plant Pest Diagnostics Center, California Department of Food and Agriculture, 3294 Meadowview Road, Sacramento, CA 95832

**Keywords:** California, D2-D3 of 28S rRNA gene, Intergenic COII-16S mitochondrial gene region, Texas

## Abstract

In May 2021, the Parana coffee root-knot nematode, *Meloidogyne paranaensis* was identified using molecular markers from a potted elephant ear plant (*Caladium* sp.) originated from San Antonio, Texas, USA. This nematode was found in a mixture with the peanut root-knot nematode, *Meloidogyne arenaria.* The molecular analysis showed that the intergenic *COII*-16S gene region and the D2–D3 of 28S rRNA gene sequences allowed differentiating *M. paranaensis* from the related root-knot nematode species of the tropical group. To the best of our knowledge, it is the first report of *M. paranaensis* in the continental United States.

In May 2021, the soil sample taken from a potted elephant ear plant (*Caladium* sp.) originating from San Antonio, Texas, USA was sent for analysis in the Nematology Laboratory, Plant Pest Diagnostics Center, California Department of Food and Agriculture, Sacramento, California. Several second-stage juveniles (J2) of the root-knot nematodes (RKN) were detected in the extracts from this soil sample. The analysis of these juveniles using several molecular markers revealed that this sample contained a mixture of two root-knot nematode species: the Parana coffee root-knot nematode, *Meloidogyne paranaensis* (Carneiro et al., 1996) and the peanut root-knot nematode, *Meloidogyne arenaria* (Neal, 1889) Chitwood, 1949. To the best of our knowledge, it is the first report of *M. paranaensis* in the continental United States.

*Meloidogyne paranaensis* was first described in 1996 in the state of Paraná, Brazil (Carneiro et al., 1996; Campos and Vallain, 2005). This species is considered as one of the most destructive RKN species parasitizing coffee in Brazil and in the Americas. It has been also reported from Colombia, Costa Rica, Guatemala, and Martinique (Subbotin et al., 2021) as well as in Mexico (López-Lima et al., 2015) and Hawaii, USA (Carneiro et al., 2004).

The objective of the present study was to provide molecular characterization of *M. paranaensis* associated with an elephant ear plant (*Caladium* sp.).

## Materials and methods

### Nematode extraction and morphological examination

Nematodes were extracted using the Baermann funnel method from the soil sample taken from a potted elephant ear plant (*Caladium* sp.) originated from Texas, San Antonio. Several second-stage juveniles (J2) killed by heating were morphologically examined and photographed using an automatic Infinity 2 camera attached to a compound Olympus BX51 microscope equipped with Nomarski interference contrast.

### Molecular analysis of nematode samples

DNA was extracted from single J2 specimens using the proteinase K protocol. DNA extraction and PCR protocols were as described by Subbotin (2021). The following primer sets were used in this study: (i) the forward D2A (5′-ACA AGT ACC GTG AGG GAA AGT TG-3′) and the reverse D3B (5′-TCG GAA GGA ACC AGC TAC TA-3′) amplifying the D2–D3 expansion segments of 28S rRNA gene; (ii) the forward NAD5F2 (5′-TAT TTT TTG TTT GAG ATA TAT TAG-3′) and the reverse NAD5R1 (5′-CGT GAA TCT TGA TTT TCC ATT TTT-3′) amplifying the partial mitochondrial *nad*5 gene; (iii) the forward C2F3 (5′-GGT CAA TGT TCA GAA ATTT GTG G-3′) and the reverse R-time-MeluR2 (5′-AAA TCT TYT CCC TAA TAA TTT TTC GTA-3′) amplifying the intergenic *COII*-16S region; (iv) the forward TRANAH (5′-TGA ATT TTT TAT TGT GAT TAA-3′) and the reverse MRH106 (5′-AAT TTC TAA AGA CTT TTC TTA GT-3′) amplifying the partial mitochondrial l-rRNA gene. The new sequences were submitted to the GenBank database under accession numbers: OK044499, OK044450 (*M. paranaensis,* intergenic *COII*-16S region), OK044497, OK044498 (*M. arenaria,* intergenic *COII*-16S region), OK044496 (*M. paranaensis,* partial mitochondrial l-rRNA gene), OK042291-OK042293 (*M. paranaensis,* D2–D3 of 28S rRNA gene), OK042294-OK042296 (*M. arenaria,* D2–D3 of 28S rRNA gene), OK044504-OK044506 (*M. paranaensis, nad*5 gene), OK044501, OK044502 (*M. arenaria, nad*5 gene).

PCR with the *M. paranaensis* species specific primers: the forward par-C09F (5′-GCC CGA CTC CAT TTG ACG GA-3′) and the reverse par-C09R (5′-CCG TCC AGA TCC ATC GAA GTC-3′) as described by Randig et al. (2002) was also used to test the root-knot nematode samples.

The new sequences for each gene were aligned using ClustalX 1.83 with their corresponding published gene sequences of *M. paranaensis* and other RKN species from the tropical group (De Ley et al., 2005; Tigano et al., 2006; Humphreys-Pereira et al., 2014; Alvarez-Ortega et al., 2019; Santos et al., 2020; and others). The alignment for the *COII-*16S gene sequences were used to construct phylogenetic networks using statistical parsimony (SP) as implemented in POPART software (http://popart.otago.ac.nz) (Bandelt et al., 1999).

## Results

### Morphological study

**Figure 1:**
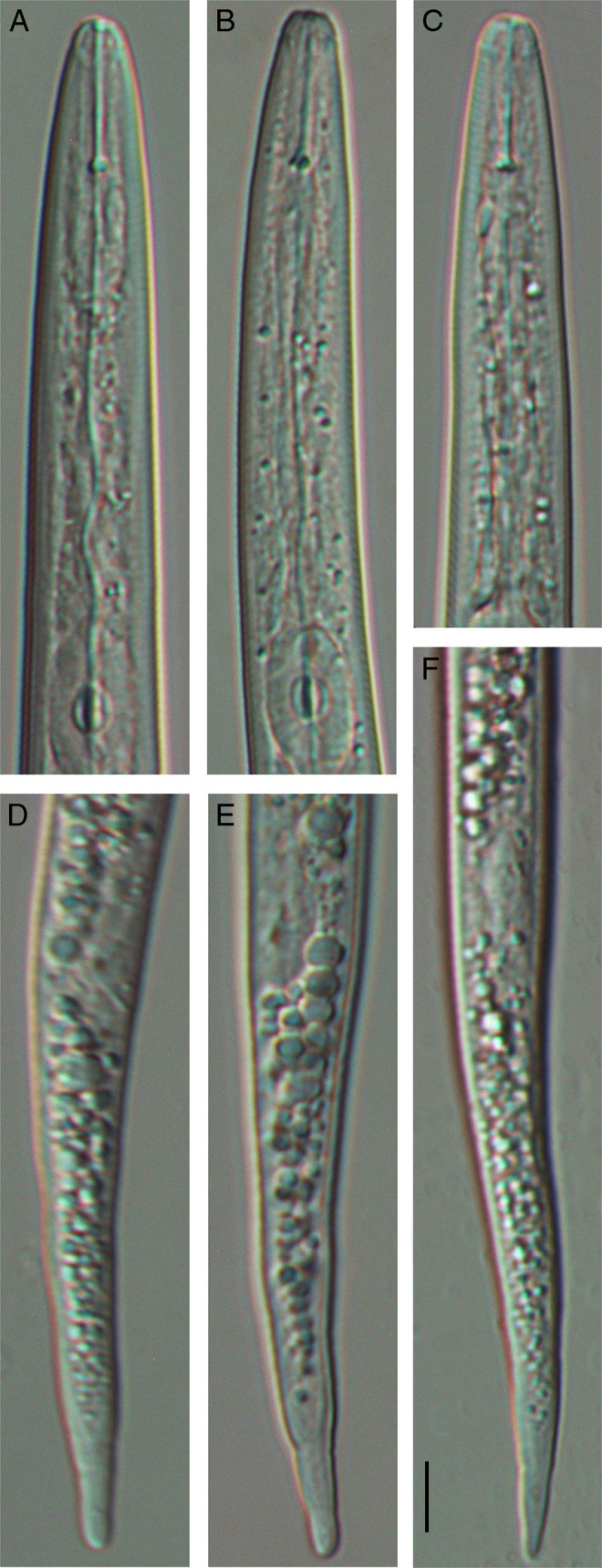
Second-stage juveniles of the root-knot nematodes extracted from a potted elephant ear plant. (A–C) Anterior region of J2s; (D–F) Posterior region of J2s. Scale = 5 *μ*m.

A few J2s were extracted from the soil sample from potted *Caladium* plant. Photos of anterior and posterior regions of J2 are given in Fig. 1. Morphology of J2s was similar with descriptions of *M. paranaensis* and *M. arenaria* (Subbotin et al., 2021).

### Molecular characterization

#### The intergenic *COII*-16S mitochondrial gene region

**Figure 2:**
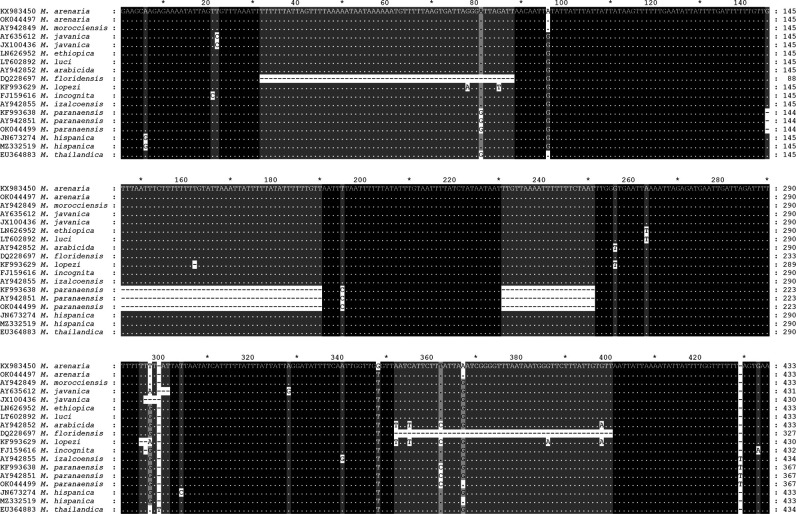
Fragment of the intergenic *COII*-16S mitochondrial gene sequence alignment (starting from 119^th^ position of the whole alignment) for *Meloidogyne* spp. from the tropical group showing two deletions in this gene region for *M. paranaensis*.

**Figure 3:**
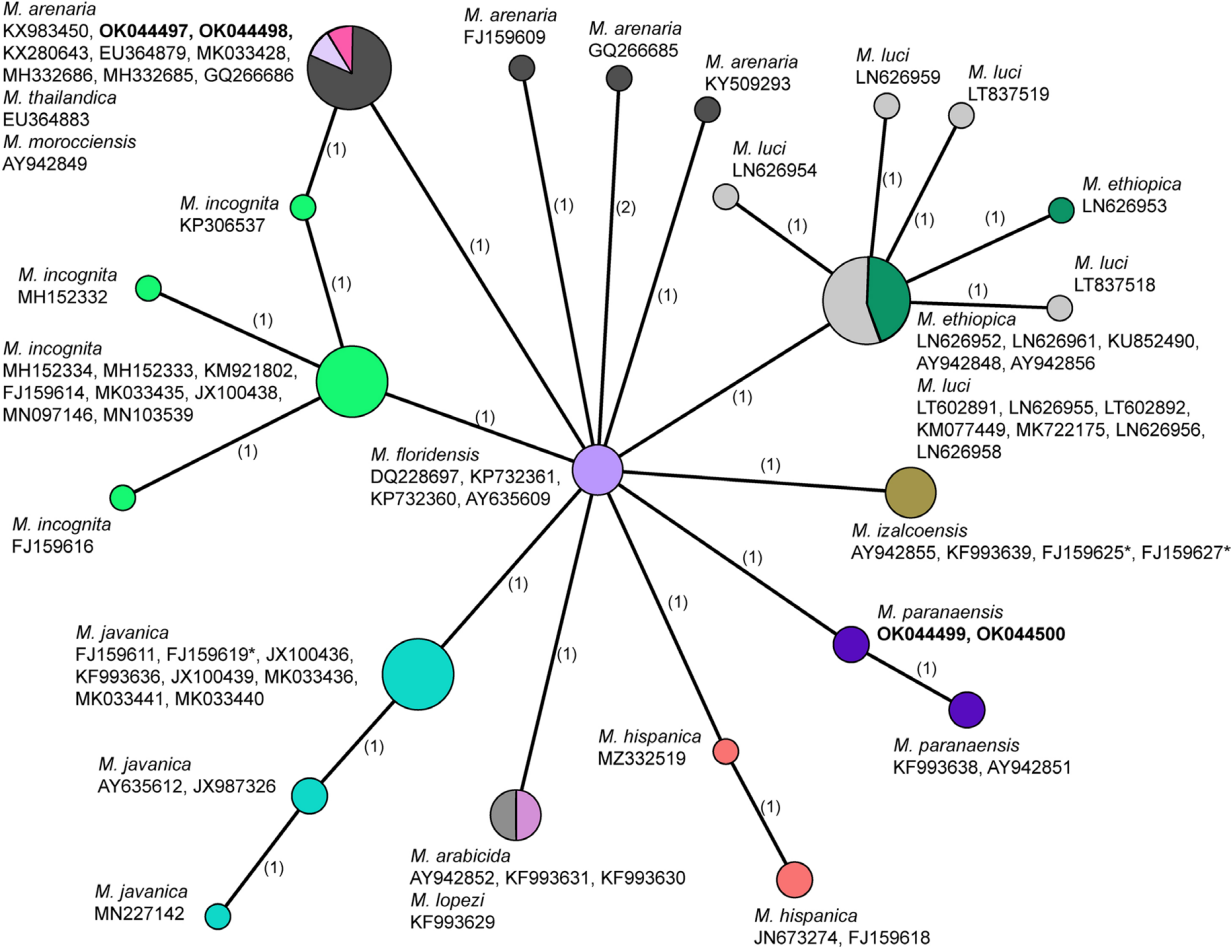
Statistical parsimony network showing the phylogenetic relationships between intergenic *COII*-16S mitochondrial gene sequences of *Meloidogyne* from the tropical group. The sequences of each species are marked by different colors. Pies (circles) represent sequences of each species with the same haplotype and their size is proportional to the number of these sequences in the samples. Numbers of nucleotide differences between the sequences are indicated on lines connecting the pies. Small black dots represent missing haplotypes. *—identified in the GenBank as *Meloidogyne incognita.*

Two new identical sequences were obtained in this study for *M. paranaensis*. The alignment was 604  bp in a length and contained 72 sequences of *Meloidogyne* species. The partial alignment is given in Fig. 2. The sequences of *M. paranaensis* contained two long deletion fragments (46  bp and 21  bp). The phylogenetic relationships of sequences of *M. paranaensis* with other the root-knot nematodes from the tropical complex reconstructed using SP are given in Fig. 3. Sequences of *M. paranaensis* from the USA were different from those from Brazil in 0.4% (2  bp).

#### The D2-D3 of 28S rRNA gene

**Figure 4:**
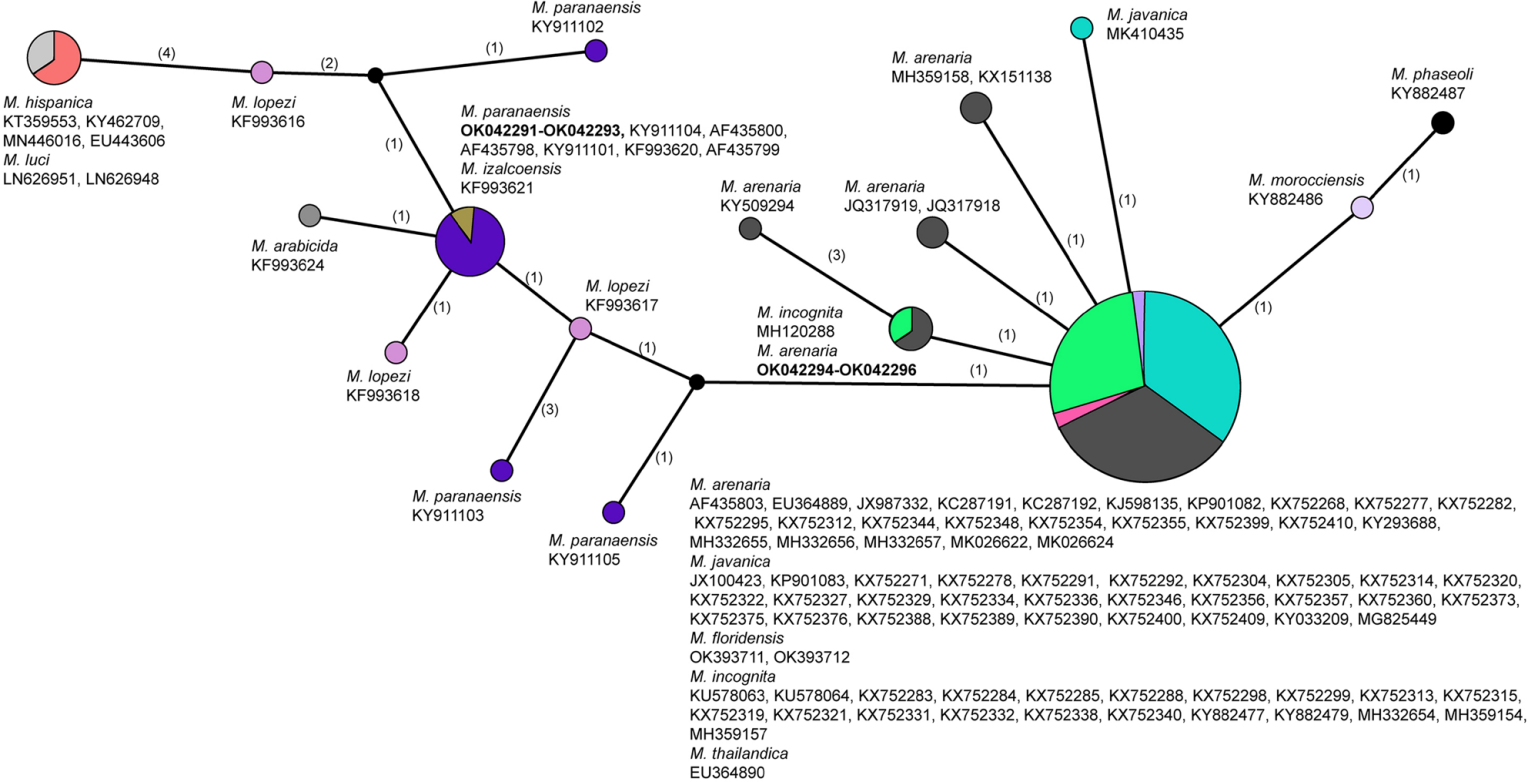
Statistical parsimony network showing the phylogenetic relationships between the D2-D3 of 28S rRNA gene sequences of *Meloidogyne* from the tropical group. The sequences of each species are marked by different colors. Pies (circles) represent sequences of each species with the same haplotype and their size is proportional to the number of these sequences in the samples. Numbers of nucleotide differences between the sequences are indicated on lines connecting the pies. Small black dots represent missing haplotypes.

Three new identical sequences were obtained in this study for *M. paranaensis*. Search of the D2–D3 of 28S rRNA gene sequences of *M. paranaensis* with Blastn in the Genbank showed 100% similarity (100% coverage) with 28S rRNA gene sequences of *M. paranaensis* (KY911101, KF993620, AF43800, AF435798) from Brazil. The alignment was 535  bp in a length and contained 112 sequences of *Meloidogyne* species. The phylogenetic relationships of sequences of *M. paranaensis* with other the root-knot nematodes from the tropical complex reconstructed using SP are given in Fig. 4.

#### The partial *nad5* gene

Three new identical sequences were obtained in this study for *M. paranaensis*. Search of *nad5* gene sequences of *M. paranaensis* with Blastn in the Genbank showed 100% similarity (100% coverage) with *nad5* sequences of identified as *M. arenaria* (MW759705-MW759707; Radoi Dumitru & Anca, unpublished) from *Pelargonium* sp., Romania. Sequences of *M. paranaensis* and *M. arenaria* (533  bp) from *Caladium* sp. differed only in one nucleotide from each other.

#### The partial mitochondrial l-rRNA gene

One new sequence was obtained in this study for *M. paranaensis*. Search of l-rRNA gene sequence of *M. paranaensis* with Blastn in the Genbank showed 99.6% similarity (100% coverage) with mitochondrial gene sequences of *M. arenaria* (KX962313; Akyazi et al., unpublished).

#### PCR with specific primer

Conventional PCR with the *M. paranaensis* species specific primers designed by Randig et al. (2002) did not generate any amplicons with DNA sample of *M. paranaensis* obtained from *Caladium* plant (data not shown).

## Discussion

The Parana coffee root-knot nematode is the most damaging species in Brazilian coffee plantations, where losses may reach 50% of the coffee yield (Carneiro et al., 1996). Although this nematode species is highly aggressive to *Coffea arabica* L., which is the primary host of this species, *M. paranaensis* has also been detected in tobacco, tomato, watermelon, several weeds and other plants (Carneiro et al., 1996; Mônaco et al., 2011; Subbotin et al., 2021; Tomaz et al., 2021). In this research we discovered *Caladium* sp. as a host plant for this nematode. Although, the plant infected with the Parana coffee root-knot nematode came from San Antonio, Texas, we cannot exclude that plant materials may have originally came from South and Central America, where *Caladium* plants, tropical perennials with colorful, heart-shaped leaves, are native to tropical forests.

Within the USA, the state of Hawaii grows their own coffee, however, California has recently established the coffee plantations in Santa Barbara and San Diego counties. Preventing the distribution of coffee pests to California will become an important task for diagnostic laboratories. Simply using the perineal pattern, J2s measurements and differential host tests could misidentify *M. paranaensis* as *M. incognita*, that has happened for years. Although analysis of rRNA and mtDNA gene sequences allows reliably identifying RKN species, new rapid, and cheap molecular diagnostic tools are needed for detection of the Parana coffee root-knot nematode.
